# Notes on a Feeding Experiment to Produce Leucoencephalitis in a Horse, with Positive Results

**Published:** 1902-08

**Authors:** Tait Butler

**Affiliations:** Raleigh, N. C.


### NOTES ON A FEEDING EXPERIMENT TO PRODUCE LEUCOENCEPHALITIS IN A HORSE, WITH POSITIVE RESULTS.

By Tait Butler, V.S.,
RALEIGH, N. C.

Throughout veterinary literature, under various names, are to be found numerous references to a disease generally known in this country as "cerebro-spinal meningitis." More recently, Pearson has described outbreaks in Pennsylvania and Delaware, reported positive results from a feeding experiment, and suggested what he considers a more appropriate name — "forage poisoning."

The writer thinks he has seen this disease in both Iowa and Mississippi, and from his observations and what he has been able to learn from the literature at his command has noted the constant absence of marked macroscopic post-mortem lesions in the brain and its enveloping membranes. In fact, the absence of post-mortem lesions sufficient to account for the pronounced clinical phenomena and early death seems to be the most constant and characteristic feature of the disease.

Early in 1901, while at the Kansas Agricultural College, I also saw a number of cases of that disease which Mayo described, in 1891, as "epizootic cerebritis," and which Buckley, of Maryland, has more recently described as "acute epizootic leucoencephalitis." Moreover, during the latter part of 1901 those counties of North Carolina bordering on Albemarle and Pamlico Sounds were visited by a severe outbreak of this same disease, from which not less than 600 horses died. During the latter part of this outbreak, in the month of December, 1901, I had an opportunity to study the clinical history and post-mortem lesions of a large number of cases.

The disease described by Mayo and Buckley, and which I have seen in Kansas and North Carolina, differs very materially from the "cerebro-spinal meningitis" of veterinary literature and the "forage poisoning" of Pearson in that the macroscopic post-mortem brain lesions are constant, extensive, and extremely characteristic and confined to the white matter of the cerebrum. This constant essential difference in post-mortem lesions and the less pronounced but important differences in clinical phenomena lead me to suspect that we have, at least, two quite distinct diseases, due to different (although probably related) causative agents.

In a feeding experiment to produce a disease having no characteristic post-mortem lesions the identity of the natural and the experimentally produced diseases must be determined chiefly by the clinical observations; but in a case where the evidences of identical, extensive, and characteristic macroscopic post-mortem lesions are added there can be no reasonable doubt as to the positive nature of the results.

These introductory observations are thought necessary to prevent any misunderstanding as to the identity of the disease under consideration.

During the early spring of 1901, a Mr. Avery, living near Wakefield, Kansas, lost, within ten days, four pure-bred Percheron horses from acute leucoencephalitis. All exhibited similar symptoms, and post-mortem examinations revealed the characteristic breaking down of more or less extensive areas in the white substance of one or both cerebral hemispheres. The sanitary condition of the stable and adjacent lots was good. The water-supply came from a hillside far above the stable, and was beyond criticism. The rough forage, while not of the best quality, was clean and sweet. The grain feed for three or four weeks immediately preceeding the outbreak of the disease consisted of corn meal. The corn from which this meal was made when examined in the crib was found to be in very bad condition, probably 10 or 15 per cent. being rotten, mouldy, and worm-eaten.

On our advice the feeding of this corn was discontinued, and although the other conditions remained the same no further loss occurred. A quantity of the worst of this corn was picked out and shipped in sacks a distance of about forty miles to the Agricultural College, at Manhattan. A small quantity of the chaff blown out by the sheller, in which was a considerable amount of light and diseased corn, was also secured. It was all kept in a dry place until July 25, 1901, when the corn in the ear was ground, cob and all, preparatory to feeding.

On July 16th two healthy colts were purchased for the feeding experiment. They were of the light harness class, and about twenty-three months old. They were confined in a dry lot higher than the surrounding country, furnished with shelter that protected them from the sun and rain, and supplied with hydrant water from the town water-works.

From July 16th to July 25th, inclusive, they were each fed $1\frac{1}{2}$ kilos of ear corn or corn and cob meal twice daily.

From July 26th to August 19th, inclusive, they were each fed $1\frac{3}{4}$ kilos of the suspected corn and cob meal twice a day, and from August 3d to the 13th they received, in addition, one feed a day—mixed with the corn and cob meal—of the chaff from the sheller previously referred to, in all amounting to about 30 kilos. The supply of suspected corn and cob meal having been consumed, these colts were fed on August 20th and the morning of August 21st (three meals) a quantity of good, marketable shelled corn.

One of the colts sickened and died at 11.40 A.M., August 21st. During the first ten days of the experiment each colt received daily about 5 kilos of alfalfa hay of the previous year's crop, and for the balance of the time each had daily about 6 kilos of new bottom land prairie hay.

The writer left Kansas August 15th, and the following observations were made by Mr. A. T. Kinsley, M. S., assistant in the Veterinary Department of the Kansas Agricultural College: "The colt that died August 21st at 11.40 A.M. was apparently in his usual health at 7.15 A.M. At 8.15 A.M., having become entangled in the wire fence, he was assisted to his feet, but staggered away to the right until he came in contact with the fence again. At 11.40 A.M. he died, and the post-mortem was made at 1 P.M. The internal organs were carefully examined, but no lesions found except a few grayish-white blotches on the liver. The left cerebral hemisphere was soft to the touch, and when cut through the white matter was broken down extensively, nearly the entire hemisphere being involved. On section the right cerebral hemisphere showed only a small broken-down area."

The time which elapsed from the beginning of the feeding of this suspected corn to the death of the animal, between three and four weeks, corresponds very closely with my observations in the natural outbreaks of the disease.

